# Short-term effect of Transcutaneous Spinal Cord Stimulation in patients with multiple sclerosis: a randomized sham-controlled crossover study

**DOI:** 10.3389/fneur.2025.1618519

**Published:** 2025-08-21

**Authors:** Eira Lotta Spieker, Marie Hoffmann, Carolin Otto, Klemens Ruprecht, Lutz Harms, Thomas Schauer, Christina Salchow-Hömmen, Nikolaus Wenger

**Affiliations:** ^1^Department of Neurology, Charité-Universitätsmedizin Berlin, Berlin, Germany; ^2^Control Systems Group, Technische Universität Berlin, Berlin, Germany; ^3^SensorStim Neurotechnology GmbH, Berlin, Germany

**Keywords:** Transcutaneous Spinal Cord Stimulation (tSCS), progressive multiple sclerosis, spasticity, rehabilitation, gait

## Abstract

**Background:**

Gait deficits and leg spasticity are frequent symptoms in Primary and Secondary Progressive Multiple Sclerosis (PPMS and SPMS). Transcutaneous spinal cord stimulation (tSCS) may alleviate these symptoms through the reduction of spinal hyperexcitability. We conducted a single-center, randomized, sham-controlled clinical crossover study (German Clinical Trials Register: DRKS00023357, https://www.drks.de/search/en) in patients with PPMS and SPMS to assess the therapy effects of tSCS on spasticity and gait in the post stimulation period.

**Methods:**

Twenty participants were included in the study to receive tSCS and sham interventions on two separate study days in randomized order. Patients and examiners were blinded to the sequence allocation, which was performed using a quasi-randomized procedure to ensure balanced group sizes. The tSCS intervention consisted of biphasic pulses applied for 30 min at 50 Hz to lumbar spinal segments. Assessments were carried out before and immediately after each intervention. The primary outcome was defined as the Modified Ashworth Scale (MAS) sum score for bilateral leg spasticity. Secondary outcomes included unilateral MAS sum scores and clinical gait assessments. We used inertial sensors to monitor gait kinematics and EMG to record Posterior-Root-Muscle-Reflexes (PRM-reflex) in leg muscles.

**Results:**

Following the exclusion of two dropouts and two participants who did not reach the target intensity, sixteen participants, evenly distributed across the two intervention sequences, were included in the analysis. In comparison to sham, tSCS had a small effect on bilateral MAS sum score (effect size = −0.25, p = 0.12, CI: −5.67–0.63, for Generalized Equation Estimation), which didn't reach significance. More patients showed an improvement in stimulation condition (10 out of 16 patients) than in sham condition (7 out of 16 patients). We observed negligible effects of tSCS on clinical gait tests, kinematic parameters and PRM-reflex recruitment.

**Conclusion:**

Our results showed that tSCS had a small but no significant effect on spasticity. A reduction of spasticity did not immediately translate into an improvement of gait performance.

**Clinical trials registration:**

https://www.drks.de/search/en, identifier: DRKS00023357.

## 1 Introduction

Multiple Sclerosis (MS) is a chronic inflammatory demyelinating disease that affects the central nervous system. Due to progressive autoimmunologic damage to descending spinal cord pathways, the disease can cause increasing spasticity of the legs and gait impairments (1). Primary Progressive MS (PPMS) and Secondary Progressive MS (SPMS) present great therapeutic challenges because of limited therapy response rates (2, 3). One recently approved disease modifying treatment for PPMS is Ocrelizumab (4). Yet, Montalban et al. (5) report a persistent decrease in walking ability of 38.9% over 2.3 years under Ocrelizumab treatment. Baclofen is an antispastic medication that is widely employed for symptom control. The drug has demonstrated efficiency in reducing the frequency of spasms and clonus, while also enhancing the range of joint movement and potentially improving gait patterns (6, 7). Yet, long-term medication is not suitable for all patients due to side effects or poor tolerability (8).

Non-invasive Transcutaneous Spinal Cord Stimulation (tSCS) has first been proposed as an alternative for spasticity therapy in the field of Spinal Cord Injury (SCI) (9, 10). Despite the placement of electrodes on the skin surface, it is possible to target the activation of large-to-medium diameter proprioceptive afferents within the posterior roots of the spinal cord (11). These structures are essential for the control of locomotion and rhythm generation (12). Applying tSCS during locomotion enhanced voluntary leg movement in SCI patients (13, 14). In the post stimulation period, tSCS further reduced leg spasticity in SCI (15–17). The underlying mechanisms of this plastic carry-over effect are not fully understood. Evidence suggests that tSCS neuromodulates pre- and postsynaptic inhibition in the control of spinal spasticity (18).

Even though permanent invasive stimulation in the upper thoracic or lower cervical spine was reported to have spasticity reducing effects on patients with MS in the 1970s and 80s (19–22), this research track has not been pursued, most likely due to the introduction of pharmaceutic advancements. Yet, more recently, non-invasive tSCS has been proposed as a treatment option for spasticity in MS. Hofstoetter et al. (23) reported that tSCS can inhibit spasticity and improve walking in MS patients, with effects lasting several hours after intervention in a single-arm pilot study. In the present study, we aimed to improve levels of evidence by comparing tSCS treatment directly against a sham control in a randomized study design with blinded assessments, in patients with PPMS or SPMS. We defined the primary outcome of the study as the bilateral Modified Ashworth Scale (MAS) sum score, a rated spasticity score. Secondary outcome parameters included unilateral MAS sum scores, performance in gait tests, kinematic parameters as well as an electrophysiological parameter. We used Inertial Measurement Units (IMU) to provide a refined kinematic characterization of potential changes in gait execution. Electromyography (EMG) signals were recorded from leg muscles before and after stimulation to monitor changes in reflex activity.

## 2 Materials and methods

### 2.1 Participants

The study was approved by the Ethics Committee of Charité-Universitätsmedizin Berlin and conducted in accordance with the Declaration of Helsinki. All participants approved written informed consent. The study was registered prospectively at the German Clinical Trials Register (DRKS00023357). We included patients that were diagnosed with Primary or Secondary Progressive MS and gait deficits due to spasticity in the legs. Exclusion criteria for the study comprised active lumbosacral nerve root compression, condition after surgery of the vertebral bodies, metal implants in the stimulation area, pacemaker implant, MS relapse event in the past six months, or current pregnancy. As this was a pilot study, the sample size was based on a similar previously published study (23) in this clinical population and by the available funding and recruitment capacity. The participants maintained their medication regime during the study.

### 2.2 Study protocol

**Figure 1:**
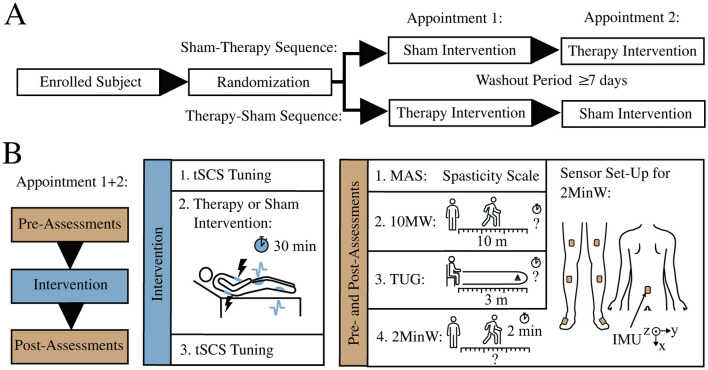
Study design and protocol. **(A)** Crossover study design with randomized order of intervention condition. **(B)** Study protocol of each appointment exists of pre-assessments, intervention, and post-assessments. Each participant conducted a sham and a therapy condition. The assessments comprise the Modified Ashworth Scale (MAS), a 10 Meter Walk Test (10MW), the Timed-Up-and-Go Test (TUG) and a 2 Minute Walk Test (2MinW). Inertial Measurement Units (IMUs) on hip, legs and feet recorded the kinematic data during the 2MinW.

The study design was comprised of two conditions, (1) a 30 min tSCS therapy session and (2) a sham stimulation in randomized order for each patient (Figure 1A). Study examiners implemented a quasi-randomization using Microsoft Excel's random number generator, ensuring balanced group sizes. Participants and outcome raters of the clinical spasticity score were blinded to the assigned sequence. Allocation concealment was maintained until after the trial. Study examiners were responsible for enrolling participants and assigning them to the intervention sequences. Between the two conditions, we ensured a washout phase of at least seven days. Figure 1B displays the study protocol at each study appointment. Assessments were performed prior (pre-therapy, pre-sham) and immediately after each intervention (post-therapy, post-sham) in a fixed order. The assessments consisted of a clinical examination of spasticity and three gait tests: the 10 Meter Walk Test (10MW), the Timed-Up-and-Go Test (TUG) and the 2 Minute Walk Test (2MinW). The total duration of each assessment battery was approximately 20 min. We determined the required time for the 10MW and TUG through recorded videos. The patients completed the gait tests with their preferred walking aid. The walking aid used in each test remained the same on both study days. The examiner instructed the gait speed as “brisk but safe”. For the 2MinW, we mounted IMUs (WaveTrack, Cometa srl, Italy) on the feet, shanks, thighs and hip to record 3D acceleration and gyroscopic data at 286 Hz for kinematic analysis.

During spasticity ratings, a blinded rater determined the Modified Ashworth Scale (MAS) for hip, knee, and ankle joints (24). Ratings were entered for twelve movements on each side: hip flexion, extension, abduction, adduction, internal and external rotation; knee flexion and extension; ankle dorsiflexion with hip and knee in a flexed position, and dorsiflexion, plantar flexion, and pronation of the ankle with hip and knee in an extended position. Each joint movement was scored as 0, 1, 1+, 2, 3, or 4 according to the severity of spasticity ranging from “no increase in muscle tone” (0) to “rigid in flexion/extension” (4). The maximum possible bilateral of the MAS sum score is 96 points.

### 2.3 Transcutaneous spinal cord stimulation

**Figure 2:**
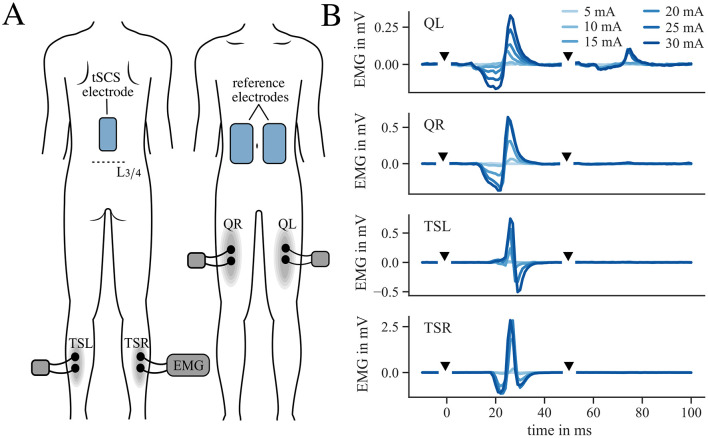
Electrode and EMG location and example of tSCS tuning. **(A)** Location of tSCS and reference electrodes as well as EMG sensor position for tuning procedure on Quadriceps (Q) and Triceps Surae (TS) muscle group on the left (L) and right (R) leg. **(B)** Example of Posterior-Root-Muscle-Reflex (PRM-reflex) responses to double pulses during tSCS tuning procedure (participant 11). Stimulation pulses are marked with black triangles.

For tSCS, we placed a self-adhesive hydrogel electrode (9 x 5 cm, axion GmbH, Leonberg, Germany) over the participant's lumbosacral spinal cord (Figure 2A), with the bottom edge 0–5 cm cranial to vertebral level L3–L4 covering the vertebrae T11-T12. The exact position depended on the individual tSCS tuning response. Two interconnected counter electrodes (8 x 13 cm) were located on the patient's lower abdomen. Throughout the interventions, the participants remained in a relaxed supine position on a daybed with a knee roll under their knees. For the stimulation, we used a current-controlled stimulator (RehaMove3, Hasomed GmbH, Germany) that delivered symmetrical biphasic rectangular pulses with 1 ms pulse-width per phase. The electrode on the patient's back served as the anode for the first phase and as the cathode for the second phase of the biphasic pulse. This configuration has been shown to elicit strong depolarization on the transition between the phases (25). Before and after the tSCS therapy or sham intervention, each patient underwent a tSCS tuning procedure to find the target therapy intensity and to investigate recruitment characteristics of leg muscles. Here, double pulses with an inter-pulse-interval of 50 ms were applied with increasing intensities starting at 5 mA and a pause of 5 s between each double pulse. To verify effective stimulation of afferent fibers, electromyographic sensors (EMG) (1,000 Hz; MuscleLab; Ergotest, Porsgrunn, Norway) recorded the responses bilaterally in two leg muscle groups (Triceps Surae (TS) and Quadriceps (Q) muscle group) (Figure 2A). A post activation depression of the Posterior-Root-Muscle-Reflex (PRM-reflex) following the second stimulation pulse confirmed the selective recruitment of posterior root afferents (26) (Figure 2B). A current level of 90% of the PRM-reflex threshold defined as the first muscle response with a peak-to-peak amplitude greater than 50 μV served as the target stimulation intensity for tSCS therapy (23). For the therapy condition, we chose a continuous stimulation of 50 Hz with a total duration of 30 min. At the beginning of the stimulation, we ramped up the current over 1-15 minutes with a median of 6 minutes depending on the participants individual comfort. The therapy application was considered successful when reaching the target therapy intensity or, if the target intensity could not be reached, but the patient reported paresthesia in the lower extremities during stimulation (16, 27, 28). For the sham condition, we applied a time limited current at maximum 20% of the therapy target for a total of 3 min. The examiner then turned off the stimulation but informed the patient that the stimulation was decreased to a lower level. For the residual 27 min of the sham therapy, the patients remained in supine position. This choice of the sham condition was intended (1) to evoke a transient mild tingling on the back, resembling the stimulation condition, (2) to be time-limited to a short period of few minutes (29, 30), and (3) to not evoke a direct recruitment of posterior root afferents at current levels far away from PRM-reflex threshold (30, 31). Before the first intervention, the examiner informed the participant that two different stimulation settings would be tested, and that the stimulation may or may not be perceived.

### 2.4 Kinematic analysis

From the IMU recordings during the 2MinW we derived kinematic variables. The kinematic evaluation is based on orientation estimation using quaternions. All body turns were excluded from the kinematic analysis. We processed the foot IMU data according to Laidig et al. (32) and determined the average of the maximum pitch angle (ankle dorsiflexion) observed in each step, calculated across all steps for each participant (in the following referred to as max pitch). The max pitch is defined as the maximum angle between the foot sole and the floor in the swing phase. To further extract the average Range of Motion (ROM) in each step of hip and knee joint across all steps (in the following referred to as ROM hip and ROM knee), we employed the data of the two adjunctive IMUs and used a heading drift correction (33). Only the ROM for flexion-extension movement during gait was considered, therefore we assumed the joints as 1D hinge joints. Due to data quality issues, we only included a subset of the patient population in the analysis for each kinematic parameter.

### 2.5 Outcome parameters

The primary outcome was defined as the bilateral MAS sum score of both legs. Since most patients showed asymmetric deficits, we additionally evaluated the MAS sum score and kinematics separately for the more and less severely affected leg. The more affected leg was determined separately for each reported parameter, and was defined as the leg with the higher average baseline MAS sum score, smaller average baseline ROM of the knee and hip, or smaller average baseline max pitch, respectively. Secondary outcomes included the unilateral MAS sum scores, gait test results (time, distance), kinematic gait parameters, and electrophysiological analysis of the tuning procedure. For this analysis, we calculated the maximum EMG response during the tuning procedure after the interventions and normalized it to the maximum EMG response observed before the interventions. This normalized value was then averaged across all active muscles and will be referred to as the EMG tuning ratio. We hypothesized that a reduction in leg spasticity has an influence on the excitability of spinal reflexes. Toward the end of the 30 min tSCS intervention, the participants responded to a pain and sensation questionnaire with items derived from the TES Comfort Questionnaire (34) and McGill Pain Questionnaire (35, 36) (Supplementary material 1).

### 2.6 Statistical analysis

We compared the change after the interventions (post-pre) between the two conditions for each participant and parameter. The difference between the short-term effect of tSCS- vs. sham-treatment was statistically evaluated using Generalized Estimation Equation (GEE) models (37) on the outcome parameters. We adjusted for baseline values, the order of intervention and appointment number. Only for the EMG tuning ratio the baseline adjustment was redundant. A two-sided significance level of α = 0.05 was used for the primary outcome (bilateral MAS sum score). We further calculated a standardized effect size *r* based on the model's result. The effect size was calculated using the treatment estimate (*GEE*_coeff(therapy)_) from our model, the standard deviation (SD) of the binary condition mapping (*x*) and the SD of the change in the respective outcome parameter (*y*_post − pre_).


(1) $$\begin{array}{l} r=GEE_{\text{coeff}\left(\text{therapy}\right)}\cdot \frac{SD\left(x\right)}{SD\left(y_{\text{post}-\text{pre}}\right)}. \end{array}$$

This value shows how strong the difference between the condition is, relative to the natural variation in the outcome parameter. The interpretation of the effect size aligns with Cohen's d (38), where values of 0.2 and above indicate a small effect, 0.5 and above a moderate effect, and 0.8 and above a large effect. All signal processing operations were implemented in Python (3.9). Statistical calculations were done in R (4.2.2). We further categorized each participant's individual treatment response in the MAS and gait test performance into different levels of improvement. For the MAS sum score, we distinguished between an deterioration, no change, a small improvement defined as a decrease in the MAS sum score of less than 1, a medium improvement defined as a decrease of at least 1 but less than 2, and a large improvement defined as a decrease of at least 2. For the gait test, we defined the level of improvements in the gait tests as stated in (23) with a large improvement defined as an increase in velocity of at least 0.05 m/s in the 10 MW (39), a decrease in time of at least 15% in the TUG (40, 41), and an increase in distance of at least 6.8 m in the 2MinW (42).

## 3 Results

**Figure 3:**
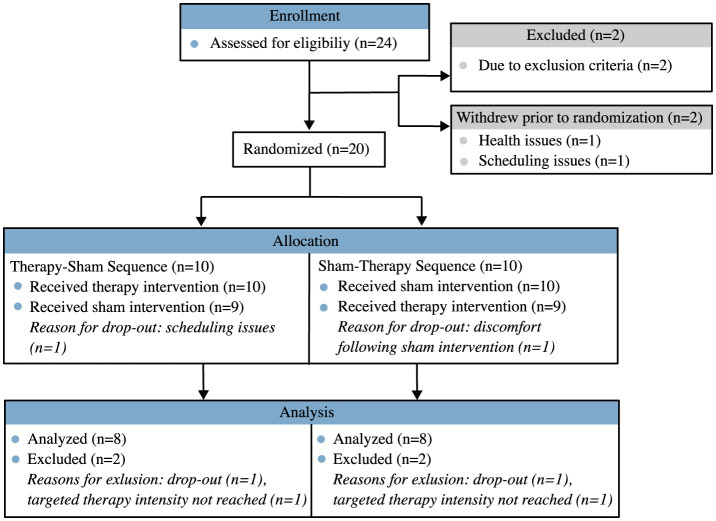
CONSORT diagram of participant recruitment.

Participants were recruited at the Department of Neurology, Charité-Universitätsmedizin Berlin, between July 2022 to January 2024. Figure 3 shows the study recruitment and allocation in a CONSORT diagram. The study concluded as planned once an adequate number of participants had been enrolled and completed both phases of the crossover trial. Out of twenty participants, we included the data of sixteen patients (54.1 ± 8.9 years, 9 male and 7 female) (Table 1). Two data sets were excluded due to dropouts and two others due to a lack of tolerance for the required stimulation intensity. The washout period varied between 7 days and 8 months with a median of 3 weeks. Baseline values did not show signs of disease progression in any of the participants during study progression. The mean baseline values in demographic and clinical variables of the two allocation groups are displayed in Table 2. An overview on the statistical results is shown in Table 3. Descriptive statistics of the primary parameter and all secondary parameters are displayed in Supplementary material 2. The mean applied stimulation intensity was 26 ± 7 mA.

**Table 1:** Demographic data of study participants at time of recruitment.

**ID**	**Diagnosis**	**Sex**	**Age**	**BMI**	**Medication**	**Walking aid during gait tests**	EDSS^*^
1	PPMS	f	59	19.3	None	Nordic walking poles	5.5
2	PPMS	m	65	23.7	unknown	None	3.5
3	SPMS	m	55	26.0	Ocrelizumab	None	5
4	SPMS	f	60	19.7	Inteferon Beta 1a	Walking stick	6.5
5	PPMS	m	58	20.1	Fampridin	None	4
6	PPMS	m	60	23.9	Ocrelizumab, Baclofen	Walking stick	6
7	PPMS	m	35	19.5	Baclofen	Walker	5
8	PPMS	f	53	19.5	None	None	6
9	PPMS	f	50	16.7	Ocrelizumab, Baclofen	Walker	6.5
10	SPMS	m	60	23.4	Baclofen	Crouches	6
11	SPMS	m	41	23.3	None	Nordic Walking poles	5
12	PPMS	f	66	25.8	Ocrelizumab	None	4
13	SPMS	f	48	25.7	Siponimod	Walker	6.5
14	PPMS	m	53	22.2	Ocrelizumab	Crouch	5.5
15	SPMS	m	42	24.3	Ocrelizumab	Walker	6
16	PPMS	f	60	24.3	Ocrelizumab, Baclofen	Walker	6.5

As patient 2 participated in a long-term placebo-controlled medication study, it remains unknown if this patient received a medication during this investigation. *EDSS estimated according to reported maximum walking distance without walking aids. EDSS, Expanded Disability Status Scale; f, female; m, male; PPMS, Primary Progressive Multiple Sclerosis; SPMS, Secondary Progressive Multiple Sclerosis.

**Table 2:** Baseline demographic and clinical characteristics of the two groups.

**Parameter**	**Sham-Therapy Sequence**	**Therapy-Sham Sequence**
Number of participants	8	8
Number of women	4	3
EDSS mean (SD)	5.19 (1.31)	5.44 (1.08)
BMI mean (SD)	23.07 (2.47)	21.62 (3.11)
**Appointment 1**		
Age mean (SD) in years	55 (10.2)	53.12 (7.9)
Bilateral MAS sum score mean (SD)	23.75 (9.17)	21.16 (17.31)
10MW mean (SD) in s	22.36 (27.12)	18.48 (14.58)
TUG mean (SD) in s	23.83 (24)	20.91 (14.09)
2MinW mean (SD) in m	84.71 (32.33) (n = 7)	72.31 (26.27)
**Appointment 2**		
Age mean (SD) in years	55 (10.2)	53.25 (8.01)
Bilateral MAS sum score mean (SD)	26.62 (7.47)	23.16 (9.48)
10MW mean (SD) in s	21.78 (29.02)	19.68 (22.44)
TUG mean (SD) in s	22.94 (27.11)	20.8 (19.75)
2MinW mean (SD) in m	94.29 (32.29) (n = 7)	69.19 (24.34)

Note that one female participant allocated to the Sham-Therapy sequence could not perform the 2MinW. EDSS, Expanded Disability Status Scale; MAS, Modified Ashworth Scale; 10MW, 10 Meter Walk Test; TUG, Timed-Up-And-Go Test; 2MinW, 2 Minute Walk Test.

**Table 3:** Statistical results for each analyzed parameter.

**Parameter**	**GEE coefficient (therapy)**	**CI low**	**CI high**	***P*-value**	**Effect size *r***
Bilateral MAS sum score (primary)	−2.53	−5.68	0.63	0.12	−0.25
MAS sum score more affected leg	−2.01	−4.41	0.39	0.10	−0.29
MAS sum score less affected leg	−0.52	−1.63	0.58	0.35	−0.11
Time 10MW	1.98	−1.29	5.24	0.24	0.16
Time TUG	1.90	−0.12	3.93	0.07	0.26
Distance 2MinW	−3.00	−6.37	0.37	0.08	−0.20
EMG tuning ratio	2.54	−24.92	30	0.86	0.03
Max pitch more affected leg	0.28	−1.48	2.04	0.75	0.04
Max pitch less affected leg	−0.92	−2.46	0.62	0.24	−0.16
ROM knee more affected leg	0.3	−1.44	2.04	0.74	0.05
ROM knee less affected leg	−0.22	−3.87	3.44	0.91	−0.02
ROM hip more affected leg	1.33	−2.55	5.21	0.5	0.16
ROM hip less affected leg	1.01	−0.21	2.23	0.11	0.18

The results were adjusted regarding the baseline values (except EMG tuning ratio), the influence of the order of condition and appointment number. The GEE coefficient (*GEE*_coeff(therapy)_) quantifies how much more (or less) patients improved (or worsened) under the therapy condition compared to the sham condition, based on the change from before to after the intervention (post-pre). MAS, Modified Ashworth Scale; 10MW, 10 Meter Walk Test; TUG, Timed-Up-And-Go Test; 2MinW, 2 Minute Walk Test; ROM, Range of Motion.

### 3.1 Primary outcome: bilateral Modified Ashworth Scale sum score

**Figure 4:**
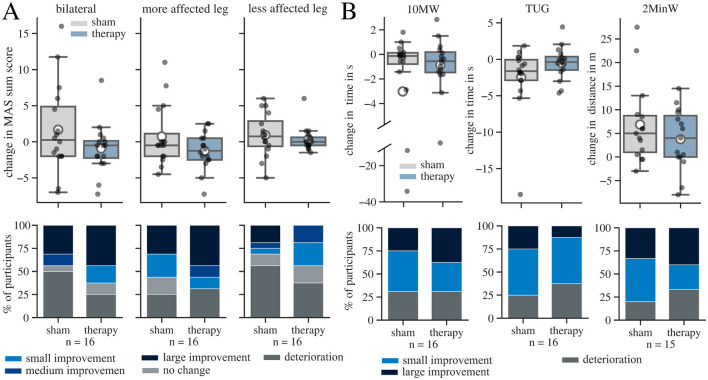
Results for changes (post-pre) in Modified Ashworth Scale (MAS) sum scores and performance in the conducted gait tests in sham and therapy condition. The whisker length in the boxplots is set to a maximum of 1.5 × the box height. Mean values are marked with a white circle. **(A)** Change in MAS sum score in both legs and in each leg individually. The more affected leg is defined as the leg with lower MAS sum score before the interventions. The percentage of participants who experienced a deterioration, no change, a small improvement (< 1), a medium improvement (≥ 1, but < 2), and a large improvement (≥ 2) in the MAS sum score are displayed underneath each boxplot in a bar chart. **(B)** Boxplots for change in gait speed during 10 Meter Walk Test (10MW) and Timed-Up-And-Go Test (TUG) and covered distance during 2 Minute Walk Tests (2MinW) in both conditions. The percentage of participants who experienced, a small improvement (increase of velocity of < 0.05 m/s in the 10MW, decrease of time < 15% in the TUG, increase of distance < 6.8 m in the 2MinW), and a large improvement (increase of velocity of ≥ 0.05 m/s in the 10MW, decrease of time ≥ 15% in the TUG, increase of distance ≥ 6.8 m in the 2MinW) are displayed underneath each boxplot in a bar chart.

The bilateral MAS sum score varied strongly among the patients (e.g., pre-therapy: mean 24, ranging from 10 to 63). The effect size of the change in bilateral MAS sum score was small (r = −0.25; 95% Confidence Interval (CI): −5.68–0.63; *p* = 0.12) (Figure 4A). The effect was statistically not significant. Although not significant, the largest numerical reduction was seen at the ankle joint (Supplementary material 3). The number of patients that improved in the MAS sum score was larger in the therapy (63%) than in the sham condition (44%). In the therapy condition, 44% of participants improved by at least 2 points, compared to 31% in the sham condition.

### 3.2 Secondary outcomes

#### 3.2.1 Unilateral Modified Ashworth Scale sum score

The statistical result for the MAS sum score of the more affected leg showed a small effect size (r = −0.29; 95% CI: −4.41–0.3; *p* = 0.1), while the absolute effect size for the less effected leg was negligible (r = −0.11; 95% CI: −1.6–0.58; *p* = 0.35) (Figure 4A). The MAS sum score of the more affected leg improved in 69% of all participants after therapy and in 56% after the sham intervention.

#### 3.2.2 Clinical gait tests

We further analyzed the difference of task duration for the 10MW and TUG and the covered distance for the 2MinW (Figure 4B). Out of the 16 participants, one patient could not complete the 2MinW due to the severity of the gait impairment. Again, the distribution of gait speed within the patient population exhibited a high variation in symptom severity at baseline (e.g. covered distance in 2MinW pre-therapy: mean of 82.6m, ranging from 19 m to 146 m). Most participants (63-80%) increased their walking speed after the intervention in all three gait tests, regardless of the applied condition. For the 10MW, the effect of tSCS is negligible compared to sham (r = 0.16; 95% CI: −1.29–5.24, *p* = 0.24). 69% of the participants improved in both conditions, but the proportion of large improvements was greater in the therapy condition (38%) compared to sham (25%). In the TUG, the number of participants, that had large improvements after the intervention, was higher in the sham (25%) than therapy (13%) condition. In the 2MinW, the proportion of patients with large improvements where similar with 31% in sham and 38% in therapy condition. The effect size of the TUG (r = 0.26; 95% CI: −0.12–3.93; *p* = 0.07) and the covered distance in the 2MinW (r = −0.2; 95% CI: −6.37–0.37; *p* = 0.08) indicate a small effect in favor of the sham condition. Overall, the results of the gait tests agree with the presence of a placebo effect.

#### 3.2.3 Gait kinematics in the 2 Minute Walk Test

**Figure 5:**
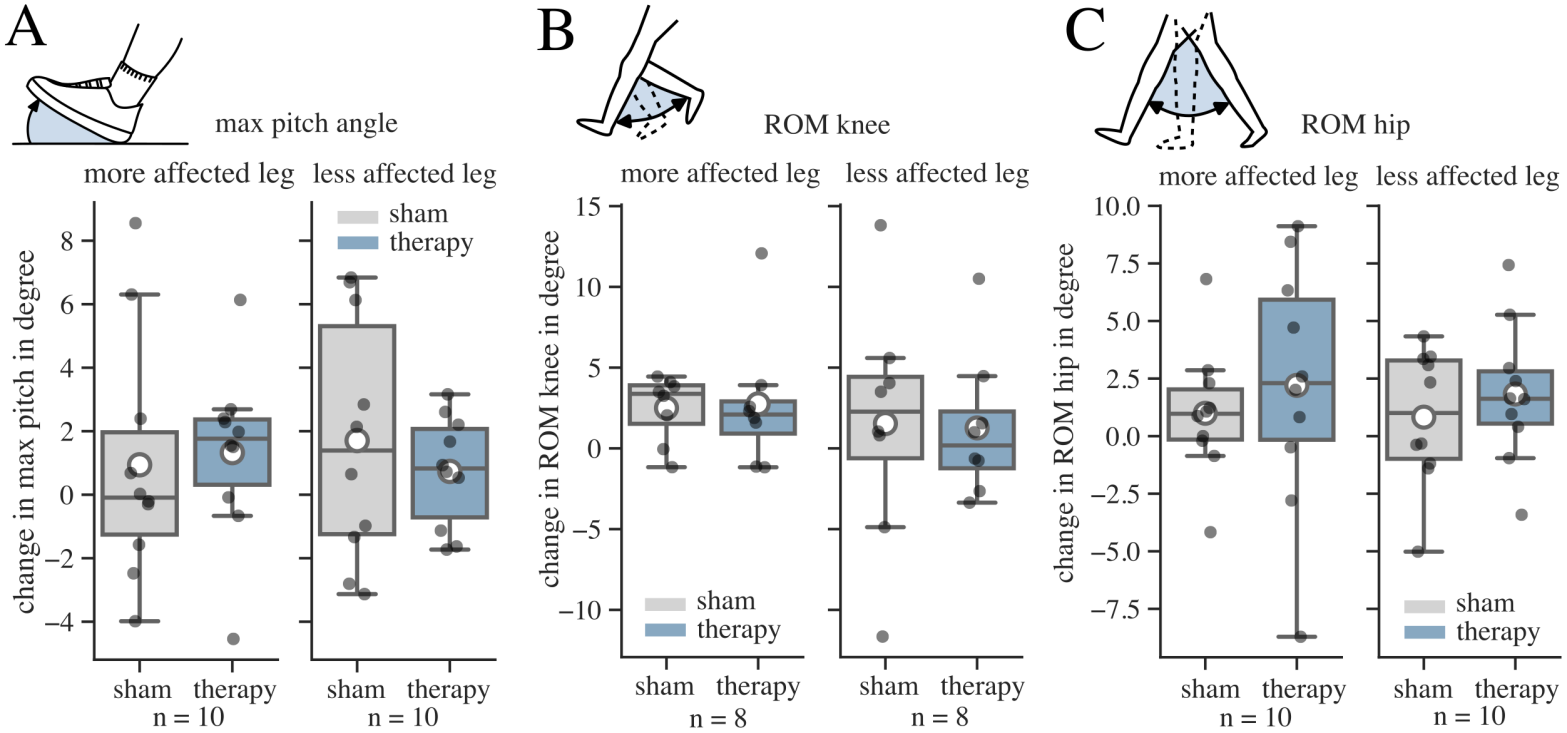
Results for change (post-pre) in kinematic variables during the 2 Minute Walk Test (2MinW) in sham and therapy condition. The whisker length in the boxplots is set to a maximum of 1.5 × the box height. Mean values are marked with a white circle. **(A)** Change in the average of the maximum pitch angle (max pitch) over all steps. The more affected leg was defined as the one with the lower max pitch angle before the interventions. **(B)** Change in the Range of Motion (ROM) of the knee joint over all steps. The more affected leg was defined as the one with the lower ROM of the knee before the interventions. **(C)** Change in the ROM of the hip joint over all steps. The more affected leg was defined as the one with the lower mean ROM of the hip before the interventions.

We further investigated kinematic variables derived from the IMU data recorded during the 2MinW (Figure 5). The effect size for the pitch angle was negligible for both legs (more affected: r = 0.04; 95% CI: −1.48–2.04; *p* = 0.75; less affected: r = −0.16; 95% CI: −2.46–0.62; *p* = 0.24) (Figure 5A). Also, the effect sizes for the ROM of the knee were negligible for both sides (more affected: r = 0.05; 95% CI: −1.44–2.04; p = 0.74; less affected: r = −0.02; 95% CI: −3.87–3.44; *p* = 0.91) (Figure 5B). For the ROM of the hip, the tSCS therapy intervention yielded an effect size of 0.16 and 0.18, respectively (more affected: r = 0.16; 95% CI: −2.55–5.21; *p* = 0.5; less affected: r = 0.18; 95% CI: −0.21–2.23; *p* = 0.11) (Figure 5C).

#### 3.2.4 Influence of tSCS on EMG recruitment curves

**Figure 6:**
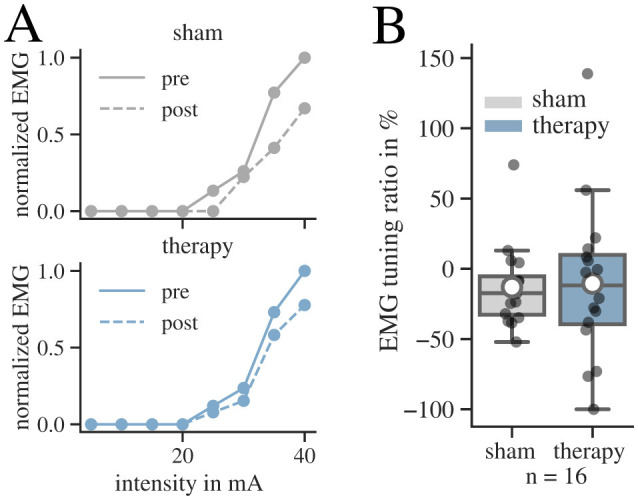
Results for the tuning procedures before (pre) and after (post) intervention for both conditions. **(A)** Recruitment curves of pre (solid) and post (dashed) state of participant 11 for the left Triceps Surae muscle are displayed for both conditions. The EMG response is measured by the peak-to-peak amplitude of the response to the first pulse at increasing stimulation intensities during the tuning procedure. The responses are normalized to the maximum EMG response recorded during the tSCS tuning before intervention. **(B)** Results of the EMG tuning ratio in the patient group for both conditions. The whisker length in the boxplots is set to a maximum of 1.5 × the box height. Mean values are marked with a white circle.

To analyze treatment-induced changes in spinal reflex activity, we determined the peak-to-peak amplitude of the PRM-reflexes before and after each intervention. An example for the normalized recruitment curves of the left TS muscle at different stimulation intensities is displayed in Figure 6A. As a measure to validate changes in response activity, we determined the EMG tuning ratio between the maximum tuning response pre and post intervention and averaged these values for the active muscles (Figure 6B). We only found a negligible effect of tSCS on the change in the EMG tuning ratio compared to sham (r = 0.03; 95% CI: −24.92–30; *p* = 0.86).

#### 3.2.5 Results of subjective questionnaires on pain and sensation

**Figure 7:**
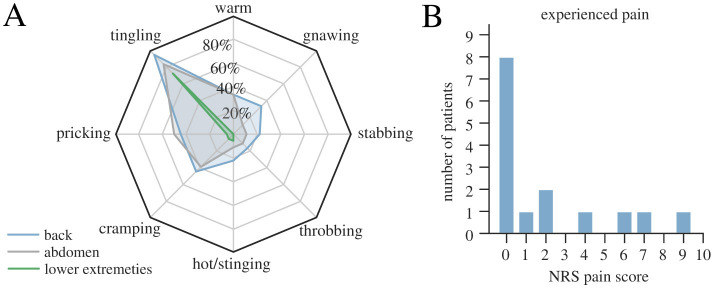
Results of the subjective pain and sensation questionnaire in therapy condition conducted toward the end of the tSCS stimulation. **(A)** Percentage of patients that experienced specific sensations during tSCS therapy application at affected body parts. **(B)** Distribution of the responses to the maximum pain score (with 0 indicating no pain at all and 10 maximum pain level).

The most prominent sensation was tingling in the back followed by tingling in the abdomen (Figure 7A). Sensations in the lower extremities were less prominent. Most participants experienced no or little pain and rated the sensations as pleasant (Figure 7B). Two patients reported high scores (7 and 9), because of experienced sudden stabbing pain around the tSCS electrode during therapy. This pain lasted a few seconds until the examiner decreased the intensity. After 1–2 min, the intensity was adjusted again to the target intensity, without any further complications. Another participant reported burning back pain and vertigo that occurred after the therapy appointment. The pain subsided within four days, and the patient experienced no lasting symptoms.

One patient decided to withdraw from the study after the sham-appointment due to experienced discomfort following the session.

## 4 Discussion

We conducted a randomized sham-controlled pilot study to investigate the short-term effect of a 30 min tSCS treatment on patients with progressive MS. The tSCS intervention showed a small effect (r = −0.25) on the bilateral MAS sum score when compared to sham, which did not reach significance. The mean bilateral MAS sum score showed a mean improvement of −0.9 ± 3.4 after therapy, while it increased by 1.7 ± 6 after the sham intervention. Gait speed across the 10MW, TUG and 2MinW did not show any clear superiority of tSCS over sham treatment. The influence of tSCS on the investigated kinematic parameters (ROM of knee and hip, max pitch) as well as the EMG tuning ratio revealed no effect. Most participants tolerated the stimulation well with no or little pain.

### 4.1 Spasticity and gait

Our results agree with the small effect sizes for spasticity reduction reported in one previous single-armed clinical study (23). The specific reduction in MAS with tSCS therapy may be of clinical interest especially for the treatment of patients showing intolerance to antispastic medication. On the other hand, we did not observe any relevant gait improvements when comparing tSCS to sham. Overall, there was also no significant correlation of the change in MAS with a change in gait speed across modalities (Supplementary material 4). Therefore, changes in leg spasticity may not immediately translate into gait improvement in patients with progressive MS. Future studies would need to address whether repeated interventions of tSCS could increase therapy effects and improve gait execution when combined with individualized physiotherapy (43), and might act on additional impairment related phenomena, such as delayed muscle soreness (44). We did observe small improvements in gait speed during the three gait tests when comparing therapy and sham conditions to their respective daily baselines. These changes were most likely explained by placebo effects and experimental factors, such as lying for 30 min in a relaxed supine position.

### 4.2 How can we define an optimal sham condition for tSCS?

Defining optimal sham conditions for the design of neuromodulation studies remains a conceptual challenge; especially in the case of transcutaneous stimulation methods such as tSCS or Transcutaneous Electric Nerve Stimulation (TENS) that can be consciously felt by the study participants. Here, we decided to apply a sham condition that provided a minimal amount of current for a short duration of 3 min. We reasoned that this intervention is appropriate because it would reduce the ability of patients to distinguish between the sham and stimulation condition. In both conditions, the patient felt a mild sensation in the back and abdomen at the beginning of the intervention. We also informed all patients that two different stimulation settings would be tested during the study, and the sensations for each intervention may differ. As described in Estes et al. (27), we informed the patient that the stimulation would be decreased to a lower level after 3 min when it was in fact turned off. These measures were influenced by previous sham-controlled studies on tSCS (29, 30, 45) or TENS (46, 47). Also, we incorporated ideas from previous studies that used sham stimulation currents restricted in time and at currents several factors under sensory threshold (31, 46, 48, 49). In our case, the sham condition was useful for detecting specific effects of tSCS on the reduction of the MAS sum score.

### 4.3 Finding optimal stimulation parameters for tSCS

A further challenge for tSCS application is to find optimal stimulation parameters in different disease modalities and patients. For spasticity treatment, previous studies in MS and SCI patients have used various frequencies (e.g. 30 Hz, 50 Hz, 5 Hz), various amplitudes, stimulation durations (30-120 min), and waveform characteristics (monophasic or biphasic) (10, 50, 51). In our case, we chose a set of parameters at 50 Hz with biphasic pulses that has previously been reported to reduce leg spasticity (16, 23). For movement control or stimulation during motor tasks the frequency is typically set to a lower value of around 30 Hz (30, 50, 52). An accompanying reduction in spasticity has been reported for these frequencies concerning the upper extremities (50). Some studies have incorporated high-frequency carrier frequencies (at 10 kHz), but their benefits for spasticity reduction remain unconfirmed (51, 53). Keesey et al. (54) even found an undesirable recruitment shift toward efferent fibers with carrier frequencies. In summary, further studies are needed to investigate optimal tSCS settings. MS patients typically show substantial heterogeneity in lesion location and symptom manifestations. Future characterizations of patient phenotypes in relation to stimulation effects could lead to the development of better personalized treatment strategies.

### 4.4 Prescribing tSCS or TENS?

Current guidelines on non-medication treatments for spasticity include TENS. In comparison, tSCS is a rather new intervention and has not entered common medical guidelines. At present, the question remains how tSCS and TENS compare. Both interventions target sensory afferents (55). TENS electrodes are typically located on the affected peripheral muscle or nerve, and therefore only activate few nerve fibers (56). In comparison, Danner et al. (57) computationally predicted that lumbar tSCS activates the roots of several spinal segments at once. The reported effect of TENS on spasticity in MS patients varies in literature. Shaygannejad et al. (58) reported a greater decrease in MAS after repeated TENS treatment compared to baclofen. Another study did not find a significant short-term effect after TENS therapy (48). Transcutaneous Spinal Cord Stimulation may present a treatment method, when several muscle groups are affected by leg spasticity or other treatment modalities are not effective.

### 4.5 Good safety profile of tSCS

Here, we used patient reported outcome measures to assess potential side effects of tSCS. Similar to previous reports (10, 13, 50, 51), our study participants reported no severe side effects. Mild side effects such as discomfort and skin irritations have been described for SCI. Six MS patients in our study reported a sensation of pain. This effect may be more prominent in MS patients with intact body sensations. In the depth of the spinal cord, tSCS primarily targets large diameter spinal afferents (12) whereas pain fibers are typically much smaller in diameter (59) and should not be directly recruited. The pain sensation was most likely explained by the activation of local pain fibers in the skin (60). In our experience, this sensation could be minimized or prevented by cleaning the skin and ensuring a homogenous contact of the stimulation electrodes with the skin surface. Overall, our data showed a good safety profile of tSCS.

### 4.6 Limitations of the study

A limitation of our study is the potential inter-rater variability of the MAS. This has been readily described in literature (61–63). Still the MAS remains one of the main primary outcome measures for rating of spasticity (63, 64). To reduce the impact of inter-rater variability in this study, we appointed the same blinded rater for all measurements in a given participant. Biomechanical assessments using isokinetic dynamometers can in principle offer complementary measurements of spasticity in future studies (65).

Another limitation of our study is the small sample size of 16 participants, which is appropriate for a pilot trial design, yet limits the statistical power and increases the risk of a type II error. The dataset establishes effects sizes and can contribute to prospective group planning. Yet, to confirm the observed effects of tSCS and establish statistical significance, a larger number of patients, ideally distributed across multiple centers, will be required in the future.

Additionally, this study investigated tSCS as a standalone intervention. Transcutaneous spinal cord stimulation paired with functional training has been demonstrated to be an effective method for spasticity reduction and functional recovery in SCI research (30, 50, 66–68) and should be considered in future studies.

As stated in the discussion, finding a suitable sham condition for tSCS is challenging. Despite the precautions taken in this study, the possibility of unblinding and it's resulting expectation bias cannot be entirely excluded.

Another limitation of the study protocol is the lack of follow-up measurements, e. g. after several hours or days after the interventions (23). Additional assessment time-points in future investigations would give additional insights on the sustainability of effects.

## 5 Conclusion

We presented the results of a randomized sham-controlled study investigating the short-term effects of tSCS on lower limb spasticity and gait in patients with PPMS and SPMS. We found a small yet not significant effect on the bilateral MAS sum score of a 30 min 50 Hz tSCS intervention compared to sham. The reduction in spasticity did not correlate with an immediate improvement in gait performance. In conclusion, the short-term reduction in leg spasticity might be of clinical interest especially for the treatment of patients showing intolerance to antispastic medication. However, for a conclusive evaluation, studies with a bigger number of participants are inevitable. Future studies could combine repeated tSCS applications with physiotherapy to further improve the therapy effects for MS.

## Data Availability

The raw data supporting the conclusions of this article will be made available by the authors, without undue reservation.
