# Supplementary material for: Short-term effect of Transcutaneous Spinal Cord Stimulation in patients with multiple sclerosis: a randomized sham-controlled crossover study
Source: Front Neurol. 2025 Aug 21;16:1618519. doi: 10.3389/fneur.2025.1618519 (PMC12408504; doi:10.3389/fneur.2025.1618519)
Supplement: Supplementary file 1 [file Data_Sheet_1.pdf]

frontiers

Supplementary Material

1 PATIENT QUESTIONNAIRES

Die Stimulation wird von jedem Anwender / jeder Anwenderin individuell wahrgenommen. Bitte teilen Sie uns mit, welche der folgenden Gefühle Sie während der Therapie bisher wahrgenommen haben.

The stimulation is perceived individually by each user. Please let us know which of the following feelings you have experienced so far during the therapy.

Beschreibung	**0 – Nicht**<br>*not*	**1 - Wenig stark**<br>*mild*	**2 - Mittel stark**<br>*medium*	**3 – Stark**<br>*strong*	Wenn > 0**wo?**<br>*if > 0: where*
Angenehm<br>*Tender*					
Wärmend<br>*Warm*					☐ Beine *legs* ☐ Rücken *back*<br>☐ Bauch *abdomen* ☐ …
Kribbeln<br>*Tingling*					☐ Beine *legs* ☐ Rücken *back*<br>☐ Bauch *abdomen* ☐ …
Brennen / heiß<br>*Hot-burning / stinging*					☐ Beine *legs* ☐ Rücken *back*<br>☐ Bauch *abdomen* ☐ …
Pochen<br>*Throbbing*					☐ Beine *legs* ☐ Rücken *back*<br>☐ Bauch *abdomen* ☐ …
Piksen<br>*Pricking*					☐ Beine *legs* ☐ Rücken *back*<br>☐ Bauch *abdomen* ☐ …
Stechen<br>*Stabbing*					☐ Beine *legs* ☐ Rücken *back*<br>☐ Bauch *abdomen* ☐ …
Verkrampft<br>*Cramping*					☐ Beine *legs* ☐ Rücken *back*<br>☐ Bauch *abdomen* ☐ …
Bohren<br>*Gnawing*					☐ Beine *legs* ☐ Rücken *back*<br>☐ Bauch *abdomen* ☐ …

Markieren Sie auf der Skala, ob Sie während der Stimulation Schmerzen empfunden haben am Bauch oder Rücken
Mark on the scale whether you felt pain in your stomach or back during the stimulation.

keine Schmerzen *no pain* |—|—|—|—|—|—|—|—|—|—| schlimmste vorstellbare Schmerzen *worst pain imaginable*

2 DESCRIPTIVE STATISTICS

Table S1: Descriptive statistics of outcome parameters. One patient could not perform the 2 Minute Walk Test due to the severity of gait impairments. Due to data quality issues, the kinematic data of some participants were excluded. MAS: Modified Ashworth Scale, 10MW: 10 Meter Walk Test, TUG: Timed-Up-And-Go Test, 2MinW: 2 Minute Walk Test, ROM: Range of Motion

	**Pre-Assessments**			**Post-Assessments**		
**Characteristic**	**Overall** (N=32)	**Sham** (N=16)	**Therapy** (N=16)	**Overall** (N=32)	**Sham** (N=16)	**Therapy** (N=16)
**Bilateral MAS (primary)**						
Mean (SD)	23.7 (11.1)	23.5 (9.0)	23.9 (13.2)	24.0 (10.8)	25.1 (7.3)	23.0 (13.5)
Median [Q1, Q3]	21.0 [17.0, 25.0]	21.8 [17.0, 24.5]	20.0 [15.9, 28.3]	23.0 [17.3, 29.0]	25.5 [19.0, 29.0]	19.3 [14.0, 29.0]
n (%)	32 (100%)	16 (100%)	16 (100%)	32 (100%)	16 (100%)	16 (100%)
**MAS more affected leg**						
Mean (SD)	13.8 (6.2)	13.9 (5.3)	13.8 (7.2)	13.6 (6.1)	14.6 (4.5)	12.5 (7.4)
Median [Q1, Q3]	11.8 [9.8, 16.3]	13.3 [9.8, 17.0]	11.4 [9.5, 16.3]	13.5 [8.8, 18.3]	15.3 [10.5, 18.8]	11.3 [6.8, 16.8]
**MAS less affected leg**						
Mean (SD)	9.8 (5.5)	9.6 (4.7)	10.1 (6.4)	10.5 (5.4)	10.5 (4.2)	10.4 (6.5)
Median [Q1, Q3]	8.5 [6.5, 11.3]	8.5 [6.8, 9.8]	9.0 [5.0, 13.8]	9.3 [6.8, 12.5]	9.8 [7.3, 12.5]	9.0 [6.0, 13.0]
**Time 10MW (s)**						
Mean (SD)	20.6 (22.8)	21.0 (24.1)	20.1 (22.3)	18.6 (18.5)	18.0 (16.4)	19.3 (20.9)
Median [Q1, Q3]	13.1 [9.9, 15.9]	12.5 [10.1, 15.2]	13.1 [9.9, 16.4]	11.8 [9.8, 15.6]	11.5 [9.9, 14.9]	12.1 [9.7, 15.6]
**Time TUG (s)**						
Mean (SD)	22.1 (20.8)	22.3 (21.3)	21.9 (20.9)	20.6 (18.8)	19.9 (17.4)	21.4 (20.6)
Median [Q1, Q3]	14.8 [12.5, 18.8]	14.1 [12.7, 18.0]	15.9 [12.4, 18.8]	14.2 [12.4, 17.0]	13.4 [12.2, 16.6]	14.8 [12.7, 17.0]
**Distance 2MinW (m)**						
Mean (SD)	79.5 (29.1)	76.4 (28.4)	82.6 (30.4)	84.8 (29.3)	83.3 (28.0)	86.3 (31.5)
Median [Q1, Q3]	78.8 [62.5, 96.0]	76.5 [60.0, 92.5]	80.0 [62.5, 100.0]	86.8 [70.5, 102.0]	83.5 [70.5, 98.0]	90.0 [67.0, 114.0]
Missing	2	1	1	2	1	1
**EMG tuning ratio (%)**						
Mean (SD)				90 (40)	90 (30)	90 (60)
Median [Q1, Q3]				80 [80, 110]	80 [70, 100]	90 [60, 110]
**Max pitch more affected leg**						
Mean (SD)	13.1 (8.0)	12.6 (7.8)	13.5 (8.6)	14.2 (9.5)	13.6 (9.2)	14.8 (10.3)
Median [Q1, Q3]	11.3 [6.7, 21.7]	11.2 [6.5, 20.6]	11.3 [7.0, 21.9]	12.1 [6.2, 23.2]	12.5 [5.9, 21.0]	11.2 [9.5, 25.0]
Excluded	12	6	6	12	6	6
**Max pitch less affected leg**						
Mean (SD)	21.2 (6.9)	20.4 (6.9)	22.0 (7.1)	22.4 (7.5)	22.1 (7.8)	22.8 (7.5)
Median [Q1, Q3]	18.4 [15.1, 28.2]	17.5 [14.7, 26.3]	23.3 [15.6, 28.7]	22.7 [14.6, 28.9]	21.0 [14.5, 27.7]	23.7 [14.7, 29.5]

Continued on next page

	Pre-Assessments			Post-Assessments		
**Characteristic**	**Overall** (N=32)	**Sham** (N=16)	**Therapy** (N=16)	**Overall** (N=32)	**Sham** (N=16)	**Therapy** (N=16)
Excluded	12	6	6	12	6	6
**ROM knee more affected leg**						
Mean (SD)	51.8 (21.4)	51.4 (22.4)	52.1 (22.0)	54.4 (20.3)	53.9 (21.8)	54.9 (20.2)
Median [Q1, Q3]	54.9 [29.5, 70.7]	55.9 [28.7, 69.6]	51.7 [32.4, 71.3]	57.4 [36.5, 71.8]	57.5 [31.9, 71.1]	52.1 [39.2, 71.8]
Excluded	16	8	8	16	8	8
**ROM knee less affected leg**						
Mean (SD)	62.7 (15.2)	62.3 (17.2)	63.1 (14.0)	64.1 (15.4)	63.8 (17.8)	64.4 (13.7)
Median [Q1, Q3]	63.8 [48.0, 73.5]	62.1 [48.0, 73.5]	63.8 [50.5, 73.0]	63.2 [53.6, 73.3]	61.0 [53.1, 76.4]	66.2 [54.9, 72.2]
Excluded	16	8	8	16	8	8
**ROM hip more affected leg**						
Mean (SD)	44.3 (11.5)	43.0 (13.2)	45.5 (10.1)	45.9 (12.0)	44.0 (13.6)	47.7 (10.6)
Median [Q1, Q3]	43.6 [34.8, 53.3]	41.6 [31.3, 52.4]	46.1 [38.4, 54.2]	47.1 [38.6, 52.4]	43.6 [33.2, 53.4]	47.9 [40.9, 51.4]
Excluded	12	6	6	12	6	6
**ROM hip less affected leg**						
Mean (SD)	55.4 (9.7)	55.6 (10.0)	55.2 (9.8)	56.7 (10.1)	56.4 (10.7)	57.0 (10.2)
Median [Q1, Q3]	58.0 [48.1, 62.4]	56.5 [47.2, 64.3]	58.0 [49.4, 61.7]	58.7 [48.1, 65.5]	57.9 [47.7, 65.8]	59.4 [48.4, 64.7]
Excluded	12	6	6	12	6	6

3 MODIFIED ASHWORTH SCALE (MAS) SPECIFIED BY JOINT

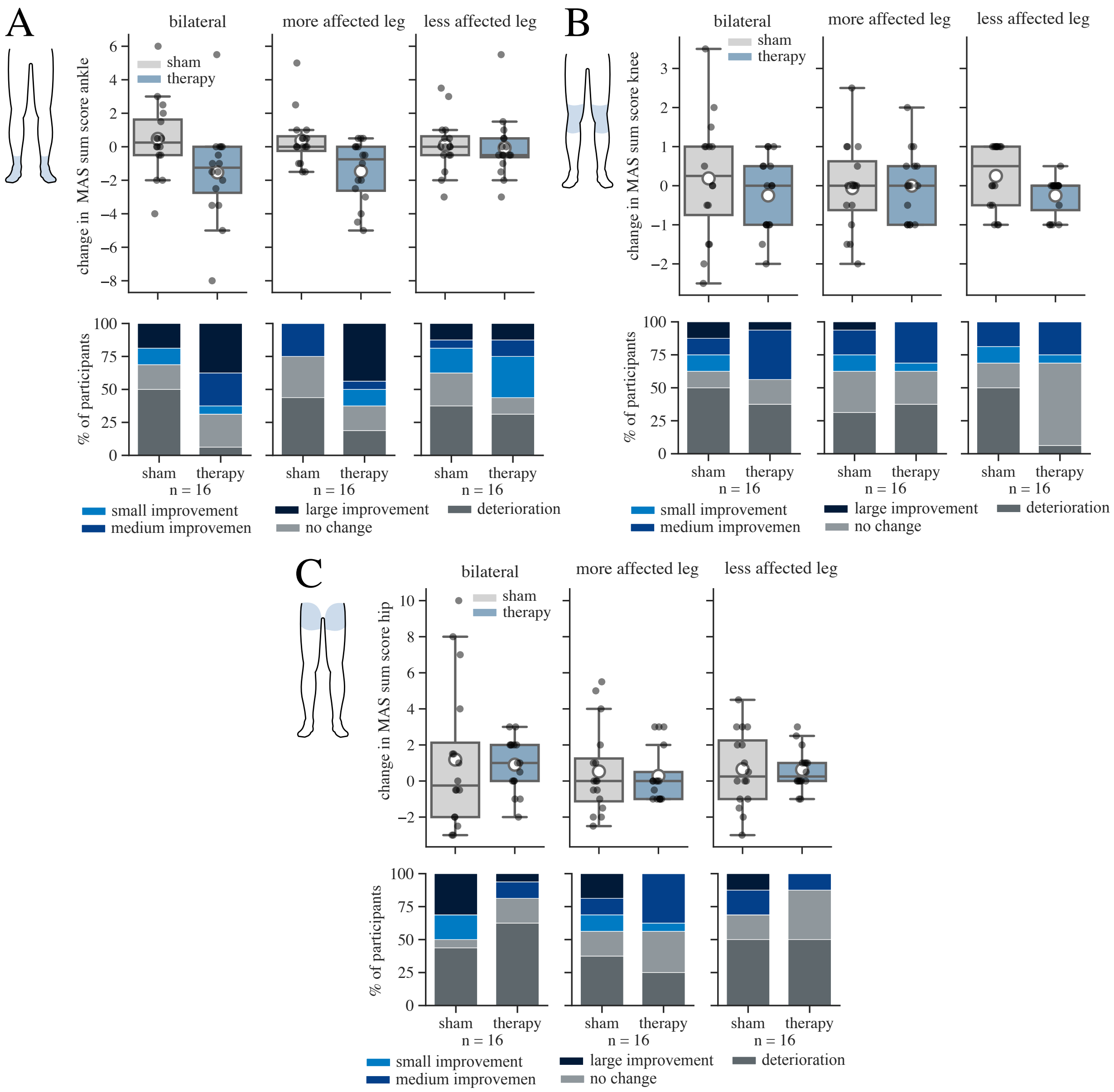

Figure S1: Results for change (post – pre) in bilateral and unilateral joint specific MAS sum scores in sham and therapy condition. The more affected leg is defined as the leg with higher MAS sum score before interventions. The whisker length in the boxplots is set to a maximum of 1.5 × the box height. Mean values are marked with a white circle. The percentage of participants who experienced a deterioration, no change, a small improvement (< 1), a medium improvement (≥ 1, but < 2), and a large improvement (≥ 2) in the MAS sum score are displayed underneath each boxplot in a bar chart.
A: Boxplots for change in MAS sum score of the ankle joint in both legs and in each leg individually.
B: Boxplots for change in MAS sum score of the knee joint in both legs and in each leg individually.
C: Boxplots for change in MAS sum score of the hip joint in both legs and in each leg individually.

4 CORRELATION OF IMPROVEMENT IN MODIFIED ASHWORTH SCALE (MAS) AND COVERED DISTANCE IN THE 2 MINUTE WALK TEST

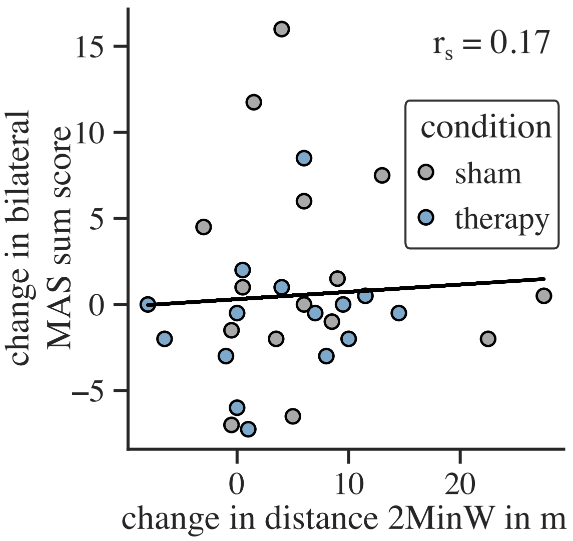

Figure S2: Correlation between change (post – pre) in bilateral Modified Ashworth Scale (MAS) sum score and covered distance in the 2 Minute Walk Test (2MinW) with Spearman's rank correlation coefficient (r_s).
